# Effects of polyploidy on the coordination of gene expression between organellar and nuclear genomes in *Leucanthemum* Mill. (Compositae, Anthemideae)

**DOI:** 10.1002/ece3.5455

**Published:** 2019-07-17

**Authors:** Christoph Oberprieler, Martina Talianova, Joachim Griesenbeck

**Affiliations:** ^1^ Evolutionary and Systematic Botany Group, Institute of Plant Sciences University of Regensburg Regensburg Germany; ^2^ Department of Biochemistry III University of Regensburg Regensburg Germany

**Keywords:** Calvin cycle, gene expression, hybridization, photosynthesis, photosystem II, polyploidy

## Abstract

Whole‐genome duplications (WGD) through polyploid speciation are associated with disruptions of well‐tuned relationships among the three plant cell genomes. Key metabolic processes comprising multi‐subunit enzyme complexes, for which partner proteins are both nuclear‐ and plastid‐encoded, are dependent on maintenance of stoichiometric ratios among the subunits to avoid cytonuclear imbalances after WGDs. By using qPCR for gene copy and transcript number quantification, we have studied the relationship of subunit expression in the two gene pairs *rbcL*/*rbcS* (the two subunits of RuBisCO) and *psbA*/*psbO* (two members of photosystem II) in closely related members of *Leucanthemum* (Compositae, Anthemideae), comprising a diploid, a tetraploid, and a hexaploid species. While gene copy numbers exhibit the expected pattern of an increase in the nuclear‐encoded partner gene relative to the plastid‐encoded one, we find that the two partner gene systems behave differently after WGD: While in the *psbA*/*psbO* partner gene system, shifts in the gene copy balance caused by polyploidization are not accommodated for through changes in transcription intensities of the two partner genes, the *rbcL*/*rbcS* system even shows an unexpected reversed dosage effect with up‐regulated transcription intensities on both the nuclear and the plastidal side. We interpret the behavior of the *psbA*/*psbO* partner gene system as being due to the stoichiometrically relaxed relationship between the two gene products caused by a fast, damage‐provoked combustion of the *psbA* gene product (the D1 core protein of PSII). Conversely, the finely tuned expression dependencies of the *rbcL*/*rbcS* system may be the reason for the observed positive feedback runaway signal as reaction to gene copy imbalances caused by a polyploidization shock.

## 1 INTRODUCTION

Considerable progress has been made in the attempt to identify effects of polyploidy on photosynthesis‐related anatomical, morphological, physiological, and biochemical traits in plants (Coate & Doyle, 2013; Warner & Edwards, 1993). However, most of the research has been focused on autopolyploids, and synthetic or young allopolyploids (Coate & Doyle, 2013). Information obtained from natural and old allopolyploids is still rather sparse, partly due to difficulties in disentangling the contribution of genome duplication, hybridization, and subsequent natural selection (Hegarty et al., 2013). During allopolyploidization, doubling of the nuclear genome occurs, yet only one set of organelles is present in the cell. Since genes involved in photosynthesis are often organized in gene networks comprising both genes located in the nucleus and genes located in the chloroplast genome, coordinated expression to produce the correct amounts of nuclear and chloroplast proteins and to maintain a proper function is required. Such coordination can occur at the level of transcription, RNA stability/degradation, translation, import, protein turnover, or metabolite flux being controlled by plastid and nuclear factors (Pogson & Albrecht, 2011). Polyploidization may thus represent a challenge with regard to maintaining the proper stoichiometry among the products of interrelated genes coming from different cellular compartments (Sharbrough, Conover, Tate, Wendel, & Sloan, 2017). This question was experimentally addressed in Coate and Doyle (2011) and Coate, Schlueter, Whaley, and Doyle (2011), who studied the evolutionary impact of polyploid and nonpolyploid duplications on genes encoding the major functional groups in photosynthesis (i.e., photosystem I, photosystem II, the light‐harvesting complex, and the Calvin cycle) in soybean (*Glycine max*), barrel medic (*Medicago truncatula*), and *Arabidopis thaliana*. In their work, the authors could observe distinct evolutionary patterns and duplicate retention in gene network evolution after duplication events: photosystem I and photosystem II gene families seem to be governed largely by dosage sensitivity, and Calvin cycle gene families seem to be less dosage sensitive and thus have larger capacities for functional divergence.

In the present study, we would like to address the question whether and how polyploidy affects the functionally tightly related gene pairs from photosystem II (PSII) and Calvin cycle (CC) in more detail. The first gene pair surveyed comprises genes encoding the large subunit (LSU) and the small subunit (SSU) of the RuBisCO (ribulose‐1,6‐bisphosphate carboxylase/oxygenase) enzyme (*rbcL* and *rbcS*, respectively). For plants, production of the right amount of RuBisCO is essential to correctly carry out photosynthesis. It is a multimeric enzyme located in the stroma of the chloroplasts (Coen, Bedbrook, Bogorad, & Rich, 1977) consisting of eight SSUs and eight LSUs. While the SSU polypeptide is encoded on nuclear genes and translated on cytoplasmic ribosomes into precursor peptides that are post‐translationally imported and processed in the chloroplast (Highfield & Ellis, 1978), the LSU is encoded by a single *rbcL* gene located on the plastidic genome. The SSU is encoded by the nucleus‐located *rbcS* gene family containing 4–10 or more genes in higher plants (Caffarri, Kouřil, Kereïche, Boekema, & Croce, 2009).

The second gene pair studied in the present contribution comprises the *psbA* and *psbO* genes from photosystem II (PSII), which is a large multi‐subunit pigment‐protein complex localized in the thylakoid membrane of plants, algae, and cyanobacteria. PSII splits water into oxygen, protons, and electrons during the photosynthetic process and thus provides energy and oxygen (Barber, 2003). The core protein D1 of the reaction center is encoded by the *psbA* gene which in higher plants usually exists as a single‐copy gene located in the plastome (Singh, 2000). The *psbO* gene encodes for the manganese‐stabilizing protein, which is in higher plants located on the lumenal side of the membrane and together with other proteins compose the oxygen‐evolving complex participating in the stabilization of the Mn‐cluster required for an efficient oxygen evolution (Popelkova & Yocum, 2011). It plays a role in the stabilization and turnover of the D1 during the PSII damage–repair cycle (Komenda, Knoppová, Krynická, Nixon, & Tichý, 2010; Yamamoto et al., 1998). In higher plants, psbO is encoded by a small nuclear gene family (Pérez‐Bueno, Barón, & García‐Luque, 2011), with one to four gene members (according to the KEGG Pathway database). A precursor protein of psbO is synthesized in the cytoplasm and imported into the chloroplasts (Keegstra, Olsen, & Theg, 1989; Popelkova & Yocum, 2011).

Our present contribution is based on representatives from the genus *Leucanthemum* (Compositae, Anthemideae), which is a polyploid complex from southern and central Europe, comprising 41 species with ploidy levels ranging from 2*x* to 22*x* (Greiner, Vogt, & Oberprieler, 2013). In the work of Greiner, Vogt, and Oberprieler (2012), Greiner et al. (2013) and Oberprieler, Greiner, Konowalik, and Vogt (2014), a closely related and geographically isolated species group from the northwest Iberian Peninsula was identified. This group was denoted as the *Leucanthemum pluriflorum* clan, and it comprises the diploid *L. pluriflorum* Pau, the tetraploid *Leucanthemum pseudosylvaticum* (Vogt) Vogt & Oberpr. (and *L. ×corunnense* Lago), and the hexaploids *Leucanthemum sylvaticum* (Brot.) Nym. and *Leucanthemum merinoi* Vogt & Castrov. We have used representatives of *L. pluriflorum* (2*x*), *L. pseudosylvaticum* (4*x*), and *L. sylvaticum* (6*x*) grown under constant conditions to (a) gain insights into the relationship of the genomic copy number and mRNA transcript abundance of functionally tightly related genes involved in photosynthesis which are located in different compartments (nucleus vs. chloroplasts) of the cell, (b) to infer chloroplast number estimates in different cell types of the leaf tissue, and (c) to interpret the results in the context of polyploidization.

**Figure 1:**
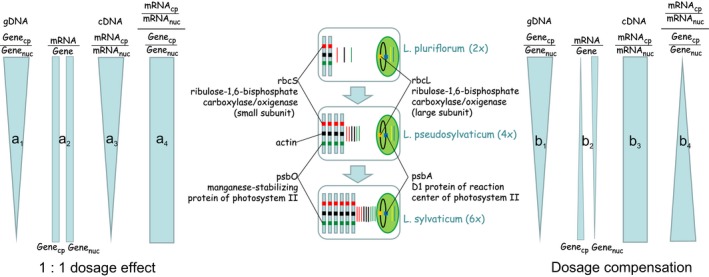
A comparison of hypothetical outcomes for gene copy (colored boxes) and transcript numbers (colored lines) of the four candidate genes under study (the two chloroplast‐encoded genes *psbA* and *rbcL* and the two nucleus‐encoded partner genes *psbO* and *rbcS*) normalized against *actin* copy numbers under the assumption of 1:1 dosage effect (left, a) and dosage compensation (right, b) after tetra‐ and hexaploidization in the *Leucanthemum pluriflorum* group. Under both scenarios, the ratio between chloroplast‐encoded gene copies (Gene_cp_) and nucleus‐encoded partner gene copies (Gene_nuc_) is expected to decrease with increasing ploidy level (*a*_1_, *b*_1_), if the doubled and tripled gene copy numbers on the nucleus side are not balanced by increased numbers of chloroplasts or plastid genomes per chloroplast. (a) Under a 1:1 dosage effect with no differences of transcription intensity (mRNA/Gene) in both genomes concerned (*a*_2_), the ratio of transcript copy numbers from chloroplast‐ and nucleus‐encoded partner genes *a*_3_ (mRNA_cp_/mRNA_nuc_) should follow the ratio between gene copy numbers (*a*_1_), and the grand index *a*_4_ measuring the discrepancy between gene copy ratio (*a*_1_) on the one hand and transcript copy ratio (*a*_3_) on the other should level out across ploidy levels. (b) Under the assumption of dosage compensation—either by an increase in transcription intensity with ploidy on the chloroplast‐gene side or by a decrease on the nucleus‐gene side (*b*_2_)—the ratio of transcript copy numbers from chloroplast‐ and nucleus‐encoded partner genes *b*_3_ (mRNA_cp_/mRNA_nuc_) should be stoichiometric, and the grand index *b*_4_ measuring the discrepancy between gene copy ratio (*b*_1_) on the one hand and transcript copy ratio (*b*_3_) on the other should increase with increasing ploidy

Figure 1 illustrates in detail the hypothetical outcomes for gene copy and transcript numbers of the four candidate genes under study expected under the assumption of a 1:1 dosage effect (no stoichiometric adjustment) after tetra‐ and hexaploidization on the one hand and dosage compensation (stoichiometric adjustment) on the other hand. Under both scenarios, the ratio between chloroplast‐encoded gene copies and nuclear‐encoded partner gene copies (*a*_1_ and *b*_1_ in Figure 1) is expected to decrease with increasing ploidy level if not the number of the latter is balanced by increased numbers of chloroplasts per cell and/or plastid genomes per chloroplast. Conversely, calculation of ratios of transcript copy numbers from chloroplast‐ and nucleus‐encoded partner genes (*a*_3_ and *b*_3_ in Figure 1) and of grand indices (*a*_4_ and *b*_4_ in Figure 1) measuring the discrepancy between gene copy ratios (*a*_1_ and *b*_1_, respectively) and transcript copy ratios (*a*_3_ and *b*_3_, respectively) would make both scenarios distinguishable.

## 2 MATERIALS AND METHODS

### 2.1 Plant material

Seeds of two geographically distinct populations for each of the three species under study (i.e., *L. pluriflorum* [2*x*], *L. pseudosylvaticum* [4*x*], and *L. sylvaticum* [6*x*]; see Table S1 for further information on the location of accessions), were grown in the greenhouse of the Institute of Plant Sciences of Regensburg University (Regensburg, Germany) under the constant light and temperature conditions. Two fully developed young rosette leaves from each plant were collected at approximately the same time (11 a.m. to 1 p.m.) during the same day (12.09.2012) and at the same developmental stage (rosette stadium), immediately put into liquid nitrogen (one leaf per Eppendorf tube) and stored at −80°C. Ten accessions for each species (five individuals for each of the two populations) were selected for further analyses.

### 2.2 DNA and RNA extraction and cDNA synthesis

Nucleic acids (RNAs and gDNA) were isolated from a single rosette leaf of the selected individuals. The extraction of total RNA was performed using a TriFast reagent (PEQLAB Biotechnologie) according to the TRIzol extraction protocol (Invitrogen). To obtain genomic DNA, DNA extraction from TRIzol using a back extraction buffer (4 M guanidine thiocyanate, 50 mM sodium citrate, 1 M Tris) was performed according to the TRIzol manufacturer's instructions (Invitrogen). Integrity of both RNA and gDNA was checked on 1.5% agarose gels. The concentration and purity of the nucleic acids were measured spectrophotometrically using the Nanodrop ND‐1000 system (Thermo Fisher Scientific). To eliminate the interference of genomic DNA in the cDNA synthesis step, a DNaseI (Fermentas) treatment of RNA was performed according to the manufacturer's recommendations followed by precipitation using 2.5 volumes of ice‐cold 96% EtOH and 0.1 volume of 3 M sodium acetate at −70°C for at least 1 hr. Conversely, the DNA extracts were treated with 1 μg/μl RNase A (Fermentas) at 37°C for 30 min, followed by ethanol precipitation. After these treatments, the quality of both, RNA and gDNA, was checked on 1.5% agarose gels and the final concentrations and purities were again measured spectrophotometrically.

The amount of 1 μg of DNaseI‐treated mRNA was used for the first‐strand cDNA synthesis using the i‐Script kit (Bio‐Rad Laboratories) according to the manufacturer's instructions. The efficiency of the reverse transcription was proved by PCR with primers specific for actin (see below for primer sequences). mRNA extracts without reverse transcriptase treatment (RT‐) were used as negative controls to check for any gDNA contamination.

### 2.3 Characterization of *Leucanthemum*‐specific PCR primers

Since the exact nucleotide sequences of the *psbA*, *psbO*, *rbcL*, and *rbcS* genes were not known for the *Leucanthemum* species under study, nucleotide sequence resources from GenBank (NCBI) had to be used to design specific primers: For the chloroplast genes *psbA* and *rbcL*, corresponding nucleotide sequences (from some Asterid representatives for *psbA* and from some Anthemideae representatives for *rbcL*) obtained from GenBank (NBCI) were aligned. Due to a high degree of conservation, specific primers could be designed in the regions of highest sequence similarity. For the nuclear *rbcS* gene, a sequence of the closely related *Chrysanthemum *×*morifolium* was available. The *C*. ×*morifolium rbcS* sequence was aligned with available sequences from other Asterids (Solanaceae representatives and *Helianthus*). Nondegenerated primers were then designed according to the *Chrysanthemum rbcS* sequence matching the most conserved regions among the aligned sequences. For the second nucleus‐encoded *psbO* gene, degenerated primers were designed based on the alignment of sequences available in GenBank. Gene‐specific primers were designed using Primer3 (Rozen & Skaletsky, 1999), and degenerated primers were designed manually. All primers are listed in Table S2. For the *actin* gene, primers Actin85F (5'‐CTGTGACAATGGAACCGGAATGG‐3') and Actin85R (5'‐TAGAAGCACTTCCTGTGGA‐3') (Steele, Guisinger‐Bellian, Linder, & Jansen, 2008) were used to amplify the corresponding sequence from all three species. PCR with gDNA templates from *L. pluriflorum*, *L. pseudosylvaticum*, and *L. sylvaticum* was performed in order to identify corresponding sequences in all species. In the case of *psbO* and *rbcS*, nested PCRs were performed. Sequences of expected length were cut from the agarose gel, purified using the PCR clean‐up gel extraction kit (Macherey‐Nagel), and either directly sequenced using the Dye Terminator Cycle Sequencing (DTCS) kit (Beckman Coulter) or cloned using the CloneJET PCR Cloning kit (Fermentas). Plasmid DNA was isolated with either the NucleoSpin Plasmid miniprep kit (Macherey‐Nagel) or the GeneJET Plasmid kit (Thermo Fisher Scientific). Inserts were then cycle‐sequenced with the DTCS kit (Beckman Coulter) using either sequence‐specific primers or cloning vector‐matching primers jetF and jetR available in the CloneJET PCR Cloning kit.

### 2.4 Quantitative real‐time PCR

To enable an absolute quantification of PCR product amounts and to test the efficiency of the PCRs, a calibration vector was constructed by ligating one copy of each gene fragment (*psba*, *psbO*, *rbcL*, *rbcS*, *actin*) into a pBluescript vector (Stratagene). In each qPCR run, three reactions, containing 100 fmol, 10 fmol, 1 fmol of the calibration vector, were coamplified in two technical replicates with the same primers besides the gDNA or cDNA templates.

A SYBR GreenI‐based methodology was used for the qPCRs. For the genes of interest, gene‐specific qPCR primers were designed matching the regions where no polymorphisms in the nucleotide sequence among the three species were present. Primer design followed the instructions in the Real‐Time PCR applications guide (Bio‐Rad Laboratories). Used primers are listed in Table S2. The qPCRs in a total volume of 20 μl were composed of 16 μl MasterMix (2 µl of 10× PCR Buffer, 0.8 µl of 25 mM MgCl_2_, 0.16 µl of 25 mM dNTPs, 0.08 µl of HotStar Polymerase [5 U/μl; all ingredients from Qiagen], 0.25 μl of 1:400,000 diluted SYBR GreenI stock [Roche], 0.40 μl of a 10 μM solution of each primer, and 11.91 μl of water) and 4.0 μl of gDNA or cDNA template. Gene copy numbers (gDNA as a template) and transcript copy numbers (cDNA as a template) were determined using the Rotor‐Gene 6000 system (Qiagen). Each reaction contained 4 ng of gDNA or 5 ng of cDNA and 4 pmol of each primer. For the RNA samples, a negative control (without addition of reverse transcriptase) was included to verify the removal of genomic DNA. The same primer pairs were used for both gDNA and cDNA. Amplification conditions were 95°C for 15 min, followed by 40 cycles at 95°C for 15 s, and 60°C for 60 s. Finally, a melting curve analysis was performed at the end of the assay to verify the amplification of a single product. Each reaction was run in a technical duplicate. Controls lacking template were included for each primer pair in every run. Data were analyzed using the Rotor‐Gene 6000 Series software, version 1.7 (Qiagen). Absolute quantification was performed using gene‐specific standard curves obtained in qPCRs with serial dilutions of the calibration vector (see above).

### 2.5 Flow cytometry

For the determination of DNA content and ploidy level, we used a two‐step flow cytometry protocol (Doležel, Greilhuber, & Suda, 2007) with *Petunia hybrida* (PxPc6) or diploid *L. pluriflorum* as an internal standard. The amount of leaf probe material (about 30 mm^2^) was approximately twice that of the internal standard material. Fragments of leaves were chopped with a razor blade in Otto I buffer (Otto, Oldiges, Göhde, & Jain, 1981). The suspension of nuclei was filtered through a mesh with a pore size of 50 μm and kept on ice, followed by the centrifugation for 5 min at 150 *g* in 4°C. The isolation buffer was removed leaving c. 50 μl above the pellet which was then dissolved in ice‐cold LB01 buffer (Doležel, Binarová, & Lcretti, 1989) with 4 mg/L of DAPI. Excitation of the sample was done using UV laser 365 nm (16 mW) with an accompanying band‐pass filter 455/50 nm on a CyFlow Space cytometer (Partec). Acquisition was automatically stopped at 8,000 nuclei. The relative genome size was calculated by multiplying the known genome size of internal standards (*P. hybrida* or *L. pluriflorum*) by the quotient between the 2C peak positions of the target probe and the internal standard in the histogram of fluorescence. The ploidy level was inferred from the relative genome sizes based on previous measurements made on plants with known chromosome numbers.

### 2.6 Confocal laser scan microscopy

Two young, fully developed leaves per accession were dissected on a manual/sledge microtome. Chloroplast numbers in guard cells, palisade, and spongy parenchyma cells were measured using a Meta Zeiss LSM 510 confocal laser scanning microscope. Excitation was performed by a 488 nm Argon laser. Chlorophyll fluorescence was detected using a long‐pass filter >650 nm. Z‐stack pictures were collected for at least eight cells of each cell type in each of the four accessions per species. Chloroplast number was determined using ImageJ v1.47 (Schneider, Rasband, & Eliceiri, 2012).

### 2.7 Data analysis

To test for a 1:1 dosage effect scenario versus a dosage compensation scenario for the studied gene pairs (Figure 1), statistical analyses were performed with the IBM SPSS Package, version 20.0 (IBM Corporation). When necessary, a transformation of the data set was performed in order to receive normally distributed variables. Analysis of variance was computed with either parametric ANOVA and post hoc tests or nonparametric Kruskal–Wallis test with subsequent Bonferroni‐corrected Mann–Whitney *U* tests.

## 3 RESULTS

To gain insight into the consequences of polyploidization in the *L. pluriflorum* clan on chloroplast number and expression patterns of photosynthesis gene pairs (“partner genes”) coded in the plastid and nuclear genomes (*psbA*/*psbO*; *rbcL*/*rbcS*), we quantified chloroplast numbers per cell (in three different cell types of the leaf) along with genomic and transcriptomic gene copy numbers in accessions representing the diploid *L. pluriflorum*, the tetraploid *L. pseudosylvaticum*, and the hexaploid *L. sylvaticum* raised in a glass house under equivalent conditions. By using a calibration vector constructed to contain single copies of the photosynthesis genes under study along with a single *actin* gene copy, it was possible to avoid a bias in analyses caused by different qPCR efficiencies in the different markers. Thus, absolute numbers for the copies of the respective gDNA or cDNA sequences contained in each experimental sample were derived. All copy numbers in the gDNA and cDNA samples were standardized against the corresponding absolute numbers derived for the *actin* gene.

### 3.1 Chloroplast number per cell

We estimated the mean number of chloroplasts in guard cells and cells of the palisade and spongy mesophyll parenchyma, respectively, throughout the three different ploidy levels (Table 1) and observed different trends in the three cell types. While hexaploids were found to contain significantly more chloroplasts in the guard cells than both diploids and tetraploids (*h* = 104.3, *df* = 2, *p* < .001; 6*x* > 4*x* = 2*x*), tetraploids were found to be significantly superior to hexaploids and those in turn significantly superior to diploids (*h* = 71.0, *df* = 2, *p* < .001; 4*x* > 6*x *> 2*x*) concerning the number of chloroplasts in cells of the palisade parenchyma. Finally, no significant differences among cytotypes were found in the cells of the spongy mesophyll parenchyma (*F* = 1.82, *df* = 2, *p* = .163; 2*x* = 4*x* = 6*x*).

**Table 1:** Chloroplast number per cell in guard cells, and cells of the palisade and spongy mesophyll parenchyma in the three *Leucanthemum* species with different ploidy level

Accession	Chloroplast number (mean ± *SD*; number of cells/number of leaves surveyed)		
	Guard cells	Palisade parenchyma cells	Spongy mesophyll cells
*Leucanthemum pluriflorum* (2*x*)			
Plu_2012_40_0_02	13.4 ± 1.2 (26/2)	125.0 ± 28.2 (31/2)	81.22 ± 29.7 (25/2)
Plu_2012_40_0_06	13.4 ± 1.5 (26/2)	95.8 ± 14.9 (26/2)	84.0 ± 25.6 (25/2)
Plu_2012_47_0_01	13.9 ± 1.3 (37/2)	103.6 ± 26.3 (25/2)	69.2 ± 18.0 (29/2)
Plu_2012_47_0_06	17.2 ± 2.3 (33/3)	143.4 ± 25.2 (25/3)	115.6 ± 35.1 (37/2)
Mean ± *SD*	14.6 ± 2.3	118 ± 30.6	89.8 ± 33.6
*Leucanthemum pseudosylvaticum* (4*x*)			
Ips_2012_02_0_04	14.7 ± 1.3 (18/2)	173.1 ± 32.1 (35/2)	107.3 ± 23.0 (22/2)
Ips_2012_02_0_07	13.6 ± 1.9 (26/3)	139.1 ± 23.3 (32/3)	84.7 ± 25.6 (33/3)
Ips_2012_16_0_03	13.9 ± 2.1 (27/2)	167.0 ± 35.4 (24/2)	83.8 ± 19.3 (31/2)
Ips_2012_16_0_10	14.2 ± 2.1 (26/2)	149.4 ± 26.7 (27/2)	82.4 ± 24.6 (30/2)
Mean ± *SD*	14.0 ± 1.9	157.2 ± 31.8	88.2 ± 24.8
*Leucanthemum sylvaticum* (6*x*)			
Syl_2012_09_0_01	14.6 ± 2.2 (17/2)	118.4 ± 25.9 (27/2)	64.9 ± 20.3 (39/2)
Syl_2012_09_0_02	16.4 ± 2.0 (19/2)	127.3 ± 22.4 (35/2)	88.8 ± 24.0 (30/2)
Syl_2012_24_0_01	19.6 ± 1.4 (38/2)	125.7 ± 24.8 (26/2)	88.4 ± 25.1 (32/2)
Syl_2012_24_0_10	17.0 ± 1.6 (43/2)	175.2 ± 42.7 (27/2)	94.6 ± 27.5 (33/2)
Mean ± *SD*	17.4 ± 2.4	136.1 ± 36.6	83.2 ± 26.7

Note

For each taxon, four individuals were analyzed and the numbers of surveyed leaves and cells are given in parentheses.

### 3.2 Genomic copy numbers

**Figure 2:**
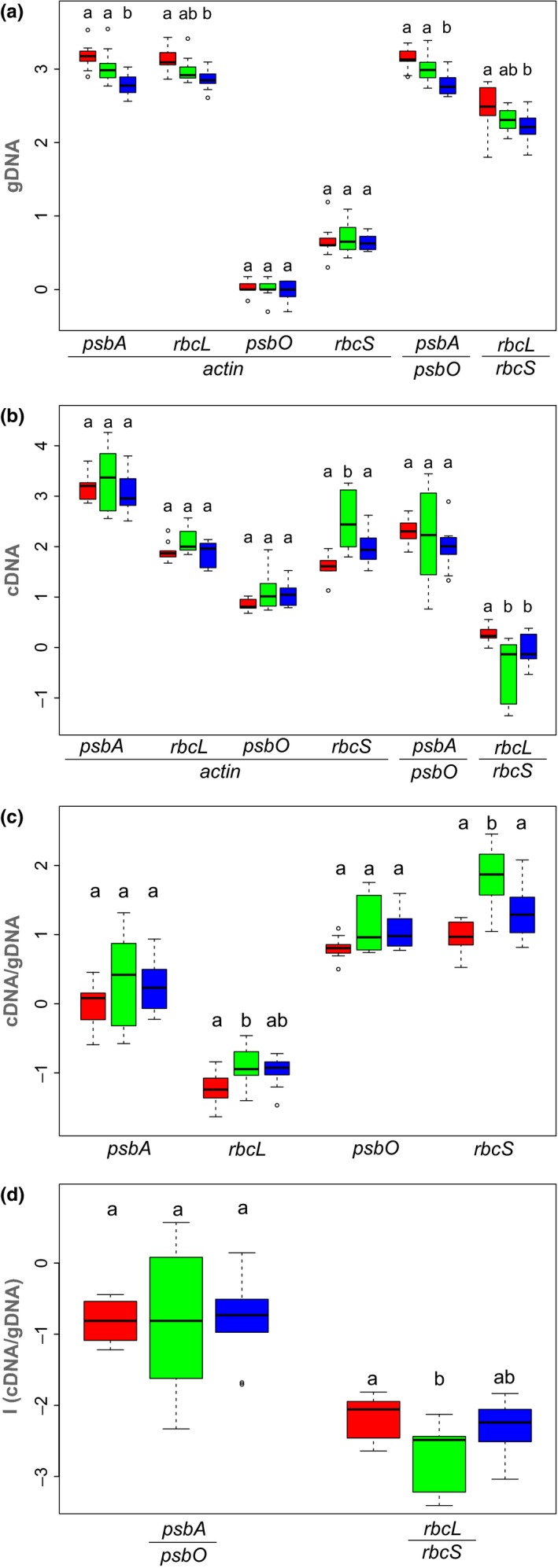
Boxplot diagrams for diploid (red), tetraploid (green), and hexaploid (blue) representatives of the *Leucanthemum pluriflorum* group studied for gene copy and transcript numbers of the two chloroplast‐encoded (cpDNA) genes *psbA* and *rbcL* and its nucleus‐encoded (nDNA) partner genes *psbO* and *rbcS*. All diagrams show a logarithmic scale on the *y*‐axis; letters above boxplots indicate statistically significant differences among the three ploidy levels (see also Table S5). (a) Results for the qPCR quantification of genomic (gDNA) copy numbers of the four genes under study; either referenced with nucleus‐encoded *actin* housekeeping gene copies (left four columns) or partner gene‐wise copy number ratio (right two columns). (b) Results for the qPCR quantification of transcript (cDNA) copy numbers for the four genes under study; either referenced with transcript numbers of the nucleus‐encoded *actin* housekeeping gene (left four columns) or partner gene‐wise transcript number ratio (right two columns). (c) Results for the qPCR‐based quantification of expression intensity calculated as the ration of transcript copy numbers (b) per gene copy numbers (a) for the four genes under study. (d) Results for the comparisons between ploidy‐dependent cpDNA‐nDNA gene copy number imbalance on the one and cpDNA‐nDNA transcript copy number imbalance on the other hand in the two partner gene systems *psbA*/*psbO* (left) and *rbcL*/*rbcS* (right)

As expected, the nucleus‐encoded partners of the two surveyed gene pairs (i.e., *psbO*, *rbcS*) showed no significant differences in copy numbers when referenced with the equally nucleus‐encoded *actin* gene copies across the ploidy levels (*psbO*: *h* = 0.567, *df* = 2, *p* = .753, 2*x* = 4*x* = 6*x*; *rbcS*: *F* = 0.144, *df* = 2, *p* = .867, 2*x* = 4*x* = 6*x*), indicating the matching increase in all three nuclear‐encoded markers through polyploidization (Figure 2a; Tables S3 and S5). While we observed gene copy numbers of *psbO* being equivalent to the number of *actin* copy numbers, however, *rbcS* showed in average an approximately fivefold higher copy number relative to *actin* gene copies. This may indicate that *rbcS* consists of more paralogous copies than the *psbO* gene. In support to the assumption that multiple paralogous *rbcS* sequences are amplified in the qPCR, melting curve analysis of DNA amplified from the gDNA resulted in a significantly broader peak than the peak obtained after amplification of *rbcS* sequence from the calibration plasmid.

In contrast to the nuclear relationships, the plastid‐encoded partners of the two surveyed gene pairs (i.e., *psbA*, *rbcL*) showed (only partly significant) ploidy‐dependent trends in copy numbers, both when referenced with the nucleus‐encoded *actin* genes (*psbA*: *F* = 10.91, *df* = 2, *p* < .001, 2*x* ≥ 4*x *> 6*x*; *rbcL*: *F* = 6.38, *df* = 2, *p* = .006, 2*x* ≥ 4*x* ≥ 6*x*) or with their nucleus‐encoded gene counterparts (*psbA/psbO*: *F* = 9.49, *df* = 2, *p* = .001, 2*x* ≥ 4*x *> 6*x*; *rbcL/rbcS*: *F* = 3.68, *df* = 2, *p* = .039, 2*x* ≥ 4*x* ≥ 6*x*). This indicates that the increase of copy numbers in the nucleus‐encoded genes caused by polyploidization is not fully compensated by an increase in copy numbers (through increase in chloroplasts per cell [compare Table 1] and/or increase in plastid genomes per chloroplast) on the plastid side. As far as gene copy ratios are concerned, therefore, our observations corroborate trends expected from the hypothetical scenarios (Figures 1, *a*_1_, *b*_1_).

### 3.3 Transcript copy numbers per cell

When compared to the expression level of the *actin* locus/loci, all photosynthesis‐related loci—irrespective of their location on the nuclear or the plastid genome—showed higher expression rates, ranging in average from around 10‐fold in *psbO* over 50‐ to 600‐fold in *rbcL* and *rbcS* to 1,600‐ to 4,800‐fold rates in *psbA* (Figure 2b; Table S4). When referenced with the expression levels of *actin*, no significant differences in expression levels of our candidate photosynthesis gene pairs were observed across ploidy levels (*psbA*: *h* = 1.30, *df* = 2, *p* = .523, 2*x* ≤ 4*x* ≥ 6*x*; *rbcL*: *h* = 3.63, *df* = 2, *p* = .163, 2*x* ≤ 4*x* ≥ 6*x*; *psbO*: *h* = 5.35, *df* = 2, *p* = .069, 2*x* ≤ 4*x* ≥ 6*x*; Table S5). An exception was the nucleus‐encoded *rbc*S gene, in which the trend of tetraploids showing higher expression rates than diploids and hexaploids gained significance (*F* = 11.98, *df* = 2, *p* < .001, 2*x* < 4*x *> 6*x*). Interestingly, a similar trend was also seen in the other genes, however, with insignificant statistical support (see above). When referenced to their nucleus‐encoded partner genes, the expression of the plastid‐encoded *rbcL* gene leveled off with its nuclear counterpart *rbcS*, while *psbA* (plastid) showed a 163‐ to 666‐fold higher expression than *psbO* (nucleus). No significant differences in these plastid versus nucleus expression ratios were found among the ploidy levels in the *psbA/psbO* gene pair (*h* = 2.30, *df* = 2, *p* = .317, 2*x* ≤ 4*x* ≥ 6*x*). In the *rbcL/rbcS* system, however, we encountered a significant higher ratio toward the plastid partner gene in diploids with only insignificant trends in tetra‐ and hexaploids (*F* = 8.72, *df* = 2, *p* = .001, 2*x* > 4*x* ≤ 6*x*). In the *rbcL/rbcS* system, therefore, the increased expression level of the nuclear gene partner (*rbcS*) caused by the higher number of gene copies through polyploidization is not fully compensated by higher expression levels (or higher copy numbers) of the plastid gene partner (*rbcL*).

### 3.4 Expression intensity relative to genomic copy numbers

Expression intensity measured as the ratio of *actin‐*normalized transcript number (cDNA) to *actin‐*normalized genomic gene copy number (gDNA) followed a trend with higher values for the nucleus‐encoded genes *rbcS* and *psbO* and lower ones for their plastid‐encoded gene partners *rbcL* and *psbA* (Figure 2c; Table S5). In all genes surveyed, there was a trend observable toward a higher expression intensity in tetraploids than in diploids and hexaploids; however, this trend became only significant in the nuclear gene *rbcS* (*F* = 13.02, *df* = 2, *p* < .001, 2*x* < 4*x *> 6*x*) and only unilaterally so in its plastidic counterpart *rbcL* (*F* = 4.13, *df* = 2, *p* = .028, 2*x* < 4*x* ≥ 6*x*). Therefore, the trend of higher expression levels of all four genes in tetraploids observed relative to the *actin* expression (Figure 2b) is caused by a higher transcription intensity in *L. pseudosylvaticum* in general and not by increased gene copy numbers. This is in accordance with the findings on the genomic level (Figure 2a), where tetraploids followed the expected trend of nucleus‐encoded gene copy numbers being equivalent to the diploids and plastid‐encoded gene copy numbers being intermediate between diploids and hexaploids. When compared with the hypothetical expectations illustrated in Figure 1, the *psbA*/*psbO* system follows the pattern of a 1:1 dosage effect (*a*_2_) in both partner genes. Conversely, in the *rbcL*/*rbcS* system, the plastidic partner gene *rbcL* shows the pattern expected under dosage compensation (*b*_2_), while the nucleus‐encoded partner gene *rbcS* either shows a 1:1 dosage compensation (*a*_2_; hexaploids) or even an unexpected overexpression (tetraploids) not covered by any of the two hypothetical scenarios.

### 3.5 Expression intensity relative to ploidy level

With the calculation of the “grand indices” *I*_(cDNA_ *_psbA/psbO_*_)/(gDNA_ *_psbA/psbO_*_)_ and *I*_(cDNA_ *_rbcL/rbcS_*_)/(gDNA_ *_rbcL/rbcS_*_)_ for each ploidy level, we tested whether the ploidy‐dependent imbalances (plastidic to nuclear copy number ratios) observed on the gDNA level (i.e., 2*x* > 4*x *> 6*x* in both gene pairs) is paralleled by corresponding trends on the cDNA level (i.e., 6*x* < 2*x* < 4*x* in *psbA/psbO*; 4*x* < 6*x* < 2*x* in *rbcL/rbcS*) across ploidy levels (Figure 2d; Table S4). For the *psbA/psbO* gene pair, we found no significant differences in this “grand index” among ploidy levels (*h* = 0.06, *df* = 2, *p* = .972; 2*x* ≤ 4*x* ≥ 6*x*), indicating that observed imbalances on the mRNA level are not significantly different from copy number differences observed on the genomic level. As a consequence, no changes in the transcriptional activities of the nuclear and plastid genes involved need to be assumed to explain the mRNA imbalances. The *psbA/psbO* system, therefore, corresponds to the hypothetical pattern expected from a 1:1 dosage effect (Figure 1, *a*_4_).

A different situation was observed in the *rbcL/rbcS* system, where the discrepancy between imbalances on the gDNA and those on the cDNA level measured by the “grand index” gained significance between the diploid and tetraploid level (*h* = 6.30, *df* = 2, *p* = .043; 2*x* > 4*x* ≤ 6*x*). This indicates that the polyploidy‐induced shift in genomic copy number relationships (with a stronger increase in copy numbers on the nuclear side relative to the plastidic side, see Figure 2a) is connected with an unproportional shift on the mRNA level. However, as indicated by the significantly lower “grand index” in the tetraploids, this shift is in a direction opposite to the expected one (where larger values in polyploids would indicate a dosage compensation scenario; Figure 1, *b*_4_). As seen before (Figure 2b,c), this is caused by an unproportional up‐regulation of the nuclear‐encoded *rbcS* gene in tetraploids relative to the (also up‐regulated) plastidic *rbcL* gene compared with diploids and hexaploids. As far as the hexaploid level is concerned, the *rbcL/rbcS* system follows the patterns observed for the *psbA/psbO* system by showing a 1:1 dosage effect.

## 4 DISCUSSION

As summarized in a recent review on cytonuclear responses to genome doubling (Sharbrough et al., 2017), there are a number of potential mechanisms to maintain cytonuclear stoichiometry in polyploid plants. Elevated nuclear gene copy numbers resulting from whole‐genome duplications (WGD) could be compensated by (a) more organelles per cell, (b) larger organelles with more genome copies per organelle, (c) increased cytoplasmic gene expression per genome copy, and (d) reduced nuclear gene expression per genome copy.

As far as compensation of duplicated nuclear gene copy numbers by increased chloroplast numbers per leaf cell (a) is concerned, the present study in the *L. pluriflorum* group found no statistically significant differences in the spongy parenchyma cells, while stomatal guard cells and cells of the palisade parenchyma exhibited a deviating pattern. Owing to the histological dominance of the palisade parenchyma, the differences observed in this tissue may be the physiologically most significant ones. Here, an expectable result of diploids containing less chloroplast per cell than polyploids was observed and corroborates similar findings of Coate et al. (2012) in the allotetraploid *Glycine dolichocarpa* when compared to its diploid progenitors *G. syndetica* and *G. tomentella*. However, the observed pattern in *Leucanthemum* is less straightforwardly interpretable than in *Glycine*. While tetraploids (*L. pseudosylvaticum*) were found having more chloroplasts per palisade parenchyma cell than diploids (*L. pluriflorum*), the hexaploids (*L. sylavticum*) exhibited significantly intermediate values (Table 1).

This result is best interpreted by assuming that the number of chloroplast per cell (and the often observed—albeit here not measured—correlated cell size) is not influenced by nucleotypic effects (effects derived from the amount of DNA per cell independent of that DNA's information content; Bennett, 1987) alone, but also by genetic effects (effects driven by changes in the information content of the genome and epigenome; Coate et al., 2012). This interpretation is all the more reasonable since we still lack a satisfying hypothesis on the genealogical relationships among the three *Leucanthemum* species involved in the present study. While sharing of the same chloroplast haplotypes across the three ploidy levels indicates that *L. pluriflorum* may have been the maternal partner in the formation of the polyploids (Greiner et al., 2013), it remained unclear whether the polyploids were formed through auto‐ or allopolyploidy and (in the latter case) which diploid could have acted as the paternal diploid (Oberprieler et al., 2014). Differences in chloroplast numbers per palisade parenchyma cells could therefore also reflect the reticulate evolutionary history of the group and its connected inheritance of the trait; with allopolyploid formation of the tetraploid *L. pseudosylvaticum* under participation of a chloroplast‐rich unknown diploid and subsequent allopolyploid formation of the chloroplast‐intermediate hexaploid *L. sylvaticum* by hybridization (back‐crossing) of the tetraploid with the chloroplast‐poor *L. pluriflorum* being only one of several conceivable pathways.

Additionally, without any experimental resynthesis of polyploids under controlled conditions, the possibility of differences in chloroplast numbers per cell being due to adaptation of the three species to different environments after their formation cannot be ruled out. With the diploid *L. pluriflorum* growing in high light‐intensity habitats of coastal vegetation types and the two polyploid species being distributed inland in semi‐shade open forest habitats or along road embankments (Vogt, 1991), these differences could also have an ecologically adaptive connotation. For the following analyses and interpretations, however, we feel justified to disregard these aspects, because all plants analyzed were cultivated under the same conditions and all quantifications of (gene and transcript) copy numbers were controlled for by internal standardizations as suggested by Coate and Doyle (2010), who also had to deal with natural diploids and polyploids with a long‐lasting history of independent evolution in *Glycine*.

Copy numbers of nuclear partner genes (i.e., *psbO*, *rbcS*) were found showing no significant increase or decrease across ploidy levels when compared to the equally nucleus‐encoded (and therefore equally duplicated) household gene *actin* (Figure 2a; Table S3). This indicates that duplication and triplication of the original (diploid) suite of copy numbers of these genes through polyploidization have not been changed dramatically after the polyploidization events. This is contrary to observations by De Smet et al. (2013) who investigated the existence of “duplication‐resistant” genes in the genomes of 20 flowering plants and found that “there is a large set of genes that is convergently restored to single‐copy status following multiple genome‐wide and smaller scale duplication events” (De Smet et al., 2013: 2898). However, in contrast to our present study group of *Leucanthemum*, for which WGD events are assumed to have occurred during the Pliocene or Pleistocene (neopolyploidization), those studied by De Smet et al. (2013) rather date back dozens to hundreds of million years (meso‐ to paleopolyploidization). Hence, stoichiometric consequences of polyploidy for cytonuclear interactions might become counterbalanced through selection for gene loss of nuclear gene families; but this seems to happen on other time‐scales than the one studied here in the *L. pluriflorum* group and may be connected to more drastic genome rearrangements observed in meso‐ and paleopolyploids (diploidization).

A further potential mechanism to maintain cytonuclear stoichiometry after WGD could be the increase in genome copies per organelle (here: chloroplast) to balance the increase in copy numbers of the nuclear partner genes (Sharbrough et al., 2017). If this was realized in the present study group, one would expect no significant differences in plastid‐to‐nucleus copy number ratios among ploidy levels. However, we observed trends in both partner gene systems that the increase in nuclear gene copies through WGD is not counterbalanced by a proportional increase in plastidic gene copies (Table S3). Together with the above‐mentioned results on chloroplast numbers per cell in our study group, this indicates that, if regulation for maintenance of cytonuclear stoichiometry after WGD takes place, this is not accommodated (exclusively) on the genomic but rather on the transcriptional level.

As far as the transcriptional level is concerned, however, our present analyses in the *L. pluriflorum* group demonstrate that the two partner gene systems under study respond differently to changes of gene copy number ratios caused by polyploidization. In the *psbA*/*psbO* system, where the chloroplast‐encoded *psbA* gene produces around 160 times more transcripts per cell than the nuclear‐encoded partner gene *psbO* in diploids (Figure 2b, Table S4) and transcription intensity (transcripts per gene copy) of *psbO* is around ten times higher than that of *psbA* (Figure 2c), transcription intensities of the two partner genes do not change significantly in polyploids (Figure 2c) and the null hypothesis of a 1:1 dosage effect could not be ruled out (Figure 2d, Table S5). In the *rbcL*/*rbcS* system, on the other hand, where absolute transcript numbers (transcripts per cell) show a shift toward higher levels of the nuclear‐encoded gene *rbcS* in polyploids (Figure 2b, Table S4) that would argue for a 1:1 dosage scenario, transcription intensity (transcripts per gene copy) of *rbcL* corresponds to a dosage compensation scenario (up‐regulation of plastid‐encoded genes to attain stoichiometry in polyploids; Figure 2c). However, the even stronger up‐regulation of the nuclear‐encoded *rbcS* partner gene (at least in tetraploids) leads to a counter‐intuitive reversed dosage effect in this gene pair (Figure 2d, Table S5).

In respect to the observed absolute transcript number differences between the nuclear and the plastidic partner genes, our findings are somewhat in line with a study of Recuenco‐Muñoz et al. (2015) who analyzed the RuBisCO system in the green algae *Chlamydomonas reinhardtii*. It is astonishing to learn that even in this ecologically and physiologically extremely important holoenzyme, the actual quantities of the two subunit proteins “have never been determined in any photosynthetic organism” (Recuenco‐Muñoz et al., 2015) until the mentioned study. Here, the authors demonstrated that *rbcL*:*rbcS* transcript and protein ratios deviated from the expected 1:1 stoichiometry and were shifted to 5:1 ratios, indicating that the small subunit (the rbcS protein) is the limiting factor for the assembly of the holoenzyme (Recuenco‐Muñoz et al., 2015). A possible reason for this shift away from the expected 1:1 ratio could be an increased transcript degradation of *rbcL* upon light as observed by Salvador, Klein, and Bogorad (1993), which would make necessary the overexpression of the plasidic partner gene to result in an equimolar ratio of gene products.

In the RuBisCO system of *Leucanthemum*, we observed a (compared with *Chlamydomonas*) less pronounced 2:1 transcript ratio in the diploids and an even more balanced 1:1 transcript ratio in the two polyploid taxa. This is in stark contrast to the 160–670:1 ratios measured in the *psbA*/*psbO* system of diploids and polyploids. As a consequence, compared with the RuBisCO system, the role of the nuclear‐encoded psbO protein as limiting factor for the assembly of the multi‐subunit pigment‐protein complex of photosystem II (PSII), in which both proteins co‐operate, appears considerably more dramatic. However, because the design of PSII allows protection for most of its protein components and damage mainly occurs at the reaction center D1 protein (coded by the *psbA* gene) through production of various radicals and active oxygen species (Baena‐González & Aro, 2002; Mulo, Sakurai, & Aro, 2012), repair of PSII requires a fast turnover of the D1 protein and thus may explain the extremely high expression level of *psbA* relative to *psbO*. As a consequence, the stoichiometric relationship between the two partner gene products psbA/psbO—being accompanied by additional 20 to 23 protein subunits in PSII (Van Bezouwen et al., 2017)—could be considered far more relaxed than the tight 1:1 one in the *rbcL*/*rbcS* system.

Indeed, the expression of rbcL and rbcS is known as being strongly correlated; both anterograde (nuclear to chloroplasts) and retrograde (plastid to nucleus) processes coordinate their production and synchronize their accumulation in photosynthetic cells (Berry, Yerramsetty, Zielinski, & Mure, 2013; Bobik & Burch‐Smith, 2015; Hauser, Popilka, Hartl, & Hayer‐Hartl, 2015; Ogawa, Suzuki, Yoshizawa, Kanno, & Makino, 2012; Suzuki & Makino, 2012). These tight expressional connections and possible disruptions to this finely tuned system caused by polyploidy might be the explanation of the astonishing observation of a somewhat reversed dosage effect in the *rbcL*/*rbcS* system. The up‐regulation of the transcription intensity of the plastidic *rbcL* gene in tetraploids (statistically significant) and hexaploids (only as a trend) relative to the diploids is in line with the pattern expected for a dosage compensation scenario, in which the photosynthetic cells try to attain stoichiometry of the two gene products rbcL and rbcS. If this increased transcription intensity on the chloroplast side, however, retrogradely leads to a comparable boost in transcription intensity on the nucleus side, the system may show a positive feedback runaway signal as reaction to gene copy imbalances caused by polyploidization. This may explain the observed different outcome for the *rbcL*/*rbcS* gene pair when compared to the more buffered *psbA*/*psbO* system.

## CONFLICT OF INTEREST

The authors declare no conflict of interests.

## AUTHOR CONTRIBUTIONS

CO and JG designed the present study; MT and JG performed the qPCR experiments and analyzed the data; CO and MT wrote the manuscript, which was improved with regard to the description of qPCR methods and in linguistic respects by JG.

## Data Availability

All data of the present study are made available through the Tables [Link], [Link], [Link], [Link], [Link].
