# Supplementary material for: Effects of polyploidy on the coordination of gene expression between organellar and nuclear genomes in Leucanthemum Mill. (Compositae, Anthemideae)
Source: Ecol Evol. 2019 Jul 17;9(16):9100–10. doi: 10.1002/ece3.5455 (PMC6706232; doi:10.1002/ece3.5455)
Supplement: Supplementary file 1 [file ECE3-9-9100-s001.doc]

**Table S1**. Information on *Leucanthemum* species, accessions, origins of plant material, and voucher material at the herbarium of the Botanic Garden & Botanical Museum Berlin-Dahlem (B) used in the present study.

| **Taxon** | **Population** | **Location (coordinates)** | **Collectors** | **Voucher** |
| --- | --- | --- | --- | --- |
| *L. pluriflorum* Pau (2*x*) | Plu2012_40 | Spain, A Coruña, Cabo Fisterra, 100 m (43.3069, -8.6186) | Greiner RH40 | B 10 0413758 |
|  | Plu2012_47 | Spain, A Coruña, Pantín, 30 m (43.6418, -8.1225) | Greiner RH47 | B 10 0413754 |
| *L. pseudosylvaticum* (Vogt) Vogt & Oberpr. (4*x*) | Ips2012_02 | Spain, Lugo, San Román, 450 m (42.8705, -7.0750) | Greiner RH2 & Hutschenreuther | B 10 0413789 |
|  | Ips2012_16 | Portugal, Viseu, Vilar, 810 m (40-9372, -7.9255 | Greiner RH16 & Greiner | B 10 0413777 |
| *L. sylvaticum* (Brot.) Nym. (6*x*) | Syl2012_09 | Portugal, Guarda, Poço da Inferno, 1000 m (40.3934, -7.5289) | Greiner RH9 & Hutschenreuther | B 10 0413782 |
|  | Syl2012_24 | Portugal, Braga, Rio Caldo – Covide, 420 m (41-7099, -8.2194) | Greiner RH24 & Greiner | B 10 0413771 |
