# Supplementary material for: Effects of polyploidy on the coordination of gene expression between organellar and nuclear genomes in Leucanthemum Mill. (Compositae, Anthemideae)
Source: Ecol Evol. 2019 Jul 17;9(16):9100–10. doi: 10.1002/ece3.5455 (PMC6706232; doi:10.1002/ece3.5455)
Supplement: Supplementary file 2 [file ECE3-9-9100-s002.doc]

**Table S2**. Sequence information for primers used for marker amplification and sequencing, along with for primers designed for qPCR and for the vector insert construction for internal standardisation of qPCR reactions.

| **Primers for PCR and sequencing** | | |
| --- | --- | --- |
| *Gene* | *Primer Name* | *5'-3' Sequence* |
| *psbA* | psbA_F1 | CATACCAAGGTTAGCACGG |
|  | psbA_R1 | GAGACGCGAAAGCGAAAGC |
| *psbO* | psbO_F1deg | ATGGCNGCNTSNYTNCAA |
|  | psbO_F2deg | GCNGGNTTYGCNCTNGCN |
|  | psbO_R1deg | RTCNGTRTCNGANGGCTG |
| *rbcL* | rbcL_F1 | TATGAAACCAAGGATACT |
|  | rbcL_R1 | AATACCGCGACTTCTATC |
| *rbcS* | rbcS_F1 | ATGGCCTCGATCTCTTCCT |
|  | rbcS_R1 | ATCGGGCTTATGTGCAATG |
|  | rbcS_R2 | ACTTGACGAACGTTGTC |
| *actin* | actin_Leu_F1 | TTCATCAAGGGAATCAGTA |
|  | actin_Leu_R1 | TGGTAGACCAAGACATACC |
|  |  |  |
| **qRT-PCR primers** | |  |
| *Gene* | *Primer Name* | *5'-3' Sequence* |
| *psbA* | psbA_qRTLeu_F1 | GGGTGCATAAGGATGTTGTG |
|  | psbA_qRTLeu_R1 | GCATATTCAGCTCCTGTTGC |
| *psbO* | psbO_qRTLeu_F1 | TCCAGGAGGTGAACGTGTG |
|  | psbO_qRTLeu_R1 | GCAACTGCATTGTCGTAACC |
| *rbcL* | rbcL_qRTLeu_F1 | TCACATGTACCCGCAGTAGC |
|  | rbcL_qRTLeu_R1 | GTCTTCGTGGTGGCcTTG |
|  | rbcL_qRTLeu_F2 | TACTACGGTACCGGCATGG |
|  | rbcL_qRTLeu_R2 | TGGCCTACTTCTTCACATCC |
|  | rbcL_qRTLeu_F3 | GCACGGTGGATGTGAAGAAG |
|  | rbcL_qRTLeu_R3 | TGCCAGAGAATTGGGAGTTC |
| *rbcS* | rbcS_qRTLeu_F1 | ACACTTGTGCGGAGTCAGTG |
|  | rbcS_qRTLeu_R1 | ACGGTTTCGTGTACCGTGAG |
| *actin* | actin_qRTLeu_F1 | TACAACGAGCTTCGTGTTGC |
|  | actin_qRTLeu_R1 | AGAAAGCACGGCCTGAATAG |
|  |  |  |
| **Primers for vector construction** | |  |
| *Gene* | *Primer Name* | *5'-3' Sequence* |
| *psbA* | SacII_psbA_F | TCCACCGCGGGGGTGCATAAGGATGTTGTG |
|  | SacII_psbA_R | GCCACCGCGGGCATATTCAGCTCCTGTTGC |
| *psbO* | BamHI_psbO_F | TAGTGGATCCTCCAGGAGGTGAACGTGTG |
|  | BamHI_psbO_R | CGGGGGATCCGCAACTGCATTGTCGTAACC |
| *rbcL* | EcoRI_rbcL_F | GCAGGAATTCTCACATGTACCCGCAGTAGC |
|  | EcoRI_rbcL_R | TATCGAATTCGTCTTCGTGGTGGCCTTG |
| *rbcS* | SalI_rbcS_F | TACCGTCGACACACTTGTGCGGAGTCAGTG |
|  | SalI_rbcS_R | CGAGGTCGACACGGTTTCGTGTACCGTGAG |
| *actin* | KpnI_actin_R | GCTGGGTACCAGAAAGCACGGCCTGAATAG |
|  | KpnI_actin_F | GCCCGGTACCTACAACGAGCTTCGTGTTGC |
