# Supplementary material for: Effects of polyploidy on the coordination of gene expression between organellar and nuclear genomes in Leucanthemum Mill. (Compositae, Anthemideae)
Source: Ecol Evol. 2019 Jul 17;9(16):9100–10. doi: 10.1002/ece3.5455 (PMC6706232; doi:10.1002/ece3.5455)
Supplement: Supplementary file 3 [file ECE3-9-9100-s003.doc]

**Table S3**. Results of qPCR analysis of gDNA gene-copy numbers of four genes *(psbA, psbO, rbcL, rbcS)* in three *Leucanthemum* species with diploid, tetraploid, and hexaploid chromosome numbers. For each species, two populations with 4-5 individuals each were analysed. Numbers given relate to gene-copy numbers either referenced to gene-copy numbers of the housekeeping gene *actin* as internal standard (first four columns) or of chloroplast-encoded gene-copy numbers relative to nucleus-encoded gene-copy numbers (last two columns).

| **Accession** | **psbA/actin** | **rbcL/actin** | **psbO/actin** | **rcbS/actin** | **psbA/psbO** | **rbcL/rbcS** |
| --- | --- | --- | --- | --- | --- | --- |
| *Leucanthemum pluriflorum* (2*x*) |  |  |  |  |  |  |
| Population 1 |  |  |  |  |  |  |
| Plu2012_40_0_02 | 1342 | 1231 | 1.0 | 4.0 | 1342.1 | 307.8 |
| Plu2012_40_0_07 | 1496 | 1141 | 0.7 | 4.9 | 2043.5 | 232.4 |
| Plu2012_40_0_04 | 1750 | 1672 | 1 | 3 | 1750.0 | 557.3 |
| Plu2012_40_0_01 | 1739 | 1429 | 1.0 | 6.0 | 1739.3 | 238.2 |
| Plu2012_40_0_06 | 948 | 973 | 1.2 | 15.5 | 812.7 | 63.0 |
| Population 2 |  |  |  |  |  |  |
| Plu2012_47_0_01 | 3393 | 2686 | 1.5 | 4 | 2262.0 | 671.5 |
| Plu2012_47_0_03 | 1275 | 1188 | 1 | 2 | 1275.0 | 594.0 |
| Plu2012_47_0_05 | 785 | 728 | 1 | 4 | 785 | 182 |
| Plu2012_47_0_06 | 1929 | 1799.5 | 1.5 | 5 | 1286.0 | 359.9 |
| **Mean (SD)** | **1629 (761)** | **1428 (576)** | **1 (0.3)** | **5 (4)** | **1477 (512)** | **356 (207)** |
|  |  |  |  |  |  |  |
| *L. pseudosylvaticum* (4*x*) |  |  |  |  |  |  |
| Population 1 |  |  |  |  |  |  |
| Ips2012_02_0_01 | 1884 | 1401 | 1.5 | 12.4 | 1235.1 | 112.9 |
| Ips2012_02_0_03 | 959 | 777 | 1.0 | 5.0 | 958.9 | 155.5 |
| Ips2012_02_0_04 | 3503 | 2587 | 1.4 | 7.7 | 2441.8 | 335.2 |
| Ips2012_02_0_07 | 749 | 754 | 0.9 | 3.7 | 845.0 | 203.3 |
| Ips2012_02_0_08 | 979 | 874 | 1.0 | 7.0 | 979.3 | 124.9 |
| Population 2 |  |  |  |  |  |  |
| Ips2012_16_0_03 | 968 | 757 | 0.5 | 3.5 | 1936.0 | 216.3 |
| Ips2012_16_0_04 | 1193 | 1043 | 1 | 3 | 1193.0 | 347.7 |
| Ips2012_16_0_07 | 760 | 654 | 1 | 4 | 760.0 | 163.5 |
| Ips2012_16_0_09 | 587 | 723 | 1.1 | 2.7 | 549.1 | 271.6 |
| Ips2012_16_0_10 | 882 | 1065 | 1.2 | 5.3 | 742.1 | 202.1 |
| **Mean (SD)** | **1246 (868)** | **1064 (580)** | **1 (0)** | **5 (3)** | **1164 (590)** | **213 (82)** |
|  |  |  |  |  |  |  |
| *L. sylvaticum* (6*x*) |  |  |  |  |  |  |
| Population 1 |  |  |  |  |  |  |
| Syl2012_09_0_01 | 606 | 703 | 0.8 | 3.3 | 763.3 | 214.9 |
| Syl2012_09_0_02 | 432 | 406 | 1.0 | 6.0 | 432.0 | 67.6 |
| Syl2012_09_0_03 | 584 | 646 | 1.3 | 4.5 | 467.5 | 143.2 |
| Syl2012_09_0_06 | 540 | 525 | 0.5 | 3.3 | 1080.4 | 161.6 |
| Syl2012_09_0_10 | 727 | 637 | 1.3 | 4.9 | 557.2 | 129.4 |
| Population 2 |  |  |  |  |  |  |
| Syl2012_24_0_01 | 1063 | 1245 | 0.8 | 3.5 | 1253.5 | 357.3 |
| Syl2012_24_0_02 | 478 | 1104 | 1.0 | 4.0 | 460.5 | 279.2 |
| Syl2012_24_0_04 | 781 | 857 | 1.1 | 5.3 | 728.8 | 162.8 |
| Syl2012_24_0_07 | 364 | 703 | 0.9 | 3.6 | 421.4 | 197.5 |
| Syl2012_24_0_10 | 780 | 758 | 1.3 | 6.7 | 585.4 | 112.9 |
| **Mean (SD)** | **635 (207)** | **758 (235)** | **1 (0)** | **4 (1)** | **675 (288)** | **183 (85)** |
