# Supplementary material for: Effects of polyploidy on the coordination of gene expression between organellar and nuclear genomes in Leucanthemum Mill. (Compositae, Anthemideae)
Source: Ecol Evol. 2019 Jul 17;9(16):9100–10. doi: 10.1002/ece3.5455 (PMC6706232; doi:10.1002/ece3.5455)
Supplement: Supplementary file 4 [file ECE3-9-9100-s004.doc]

**Table S4**. Results of qPCR analysis of cDNA transcript numbers of four genes *(psbA, psbO, rbcL, rbcS)* in three *Leucanthemum* species with diploid, tetraploid, and hexaploid chromosome numbers. For each species, two populations with 4-5 individuals each were analysed. Numbers given relate to transcript numbers either referenced to transcript numbers of the housekeeping gene *actin* as internal standard (first four columns) or of chloroplast-encoded transcript numbers relative to nucleus-encoded transcript numbers (last two columns).

| **Accession** | **psbA/actin** | **rbcL/actin** | **psbO/actin** | **rbcS/actin** | **psbA/psbO** | **rbcL/rbcS** |
| --- | --- | --- | --- | --- | --- | --- |
| *Leucanthemum pluriflorum* (2*x*) |  |  |  |  |  |  |
| Population 1 |  |  |  |  |  |  |
| Plu2012_40_0_02 | 1921 | 47 | 5 | 13 | 389.0 | 3.5 |
| Plu2012_40_0_07 | 1805 | 125 | 9 | 35 | 200.5 | 3.6 |
| Plu2012_40_0_04 | 1029 | 73 | 7 | 53 | 143.4 | 1.4 |
| Plu2012_40_0_01 | 4952 | 207 | 10 | 91 | 509.8 | 2.3 |
| Plu2012_40_0_06 | 1845 | 82 | 6 | 85 | 293.1 | 1.0 |
| Population 2 |  |  |  |  |  |  |
| Plu2012_47_0_01 | 867 | 62 | 5 | 41 | 182.6 | 1.5 |
| Plu2012_47_0_03 | 1590 | 68 | 6 | 33 | 249.4 | 2.1 |
| Plu2012_47_0_05 | 726 | 53 | 6 | 33 | 121.0 | 1.6 |
| Plu2012_47_0_06 | 811 | 78 | 10 | 46 | 77.7 | 1.7 |
| **Mean (SD)** | **1727 (1301)** | **88 (50)** | **7 (2)** | **48 (25)** | **241 (138)** | **2 (1)** |
|  |  |  |  |  |  |  |
| *L. pseudosylvaticum* (4*x*) |  |  |  |  |  |  |
| Population 1 |  |  |  |  |  |  |
| Ips2012_02_0_01 | 500 | 80 | 87 | 1809 | 5.8 | 0.04 |
| Ips2012_02_0_03 | 1239 | 87 | 54 | 1422 | 22.9 | 0.06 |
| Ips2012_02_0_04 | 947 | 103 | 14 | 86 | 68.3 | 1.20 |
| Ips2012_02_0_07 | 360 | 70 | 8 | 62 | 47.0 | 1.12 |
| Ips2012_02_0_08 | 6975 | 100 | 17 | 1327 | 418.5 | 0.08 |
| Population 2 |  |  |  |  |  |  |
| Ips2012_16_0_03 | 512 | 85 | 19 | 308 | 27.6 | 0.28 |
| Ips2012_16_0_04 | 6318 | 212 | 6 | 188 | 1148.8 | 1.13 |
| Ips2012_16_0_07 | 8990 | 200 | 6 | 372 | 1498.3 | 0.54 |
| Ips2012_16_0_09 | 4377 | 99 | 7 | 99 | 662.8 | 1.00 |
| Ips2012_16_0_10 | 18262 | 369 | 7 | 244 | 2758.9 | 1.52 |
| **Mean (SD)** | **4848 (5677)** | **140 (94)** | **22 (27)** | **591 (659)** | **666 (905)** | **1 (1)** |
|  |  |  |  |  |  |  |
| *L. sylvaticum* (6*x*) |  |  |  |  |  |  |
| Population 1 |  |  |  |  |  |  |
| Syl2012_9_0_1 | 518 | 102 | 6 | 56 | 81.2 | 1.8 |
| Syl2012_9_0_2 | 916 | 38 | 7 | 64 | 134.0 | 0.6 |
| Syl2012_9_0_3 | 1244 | 92 | 18 | 134 | 69.8 | 0.7 |
| Syl2012_9_0_6 | 322 | 33 | 15 | 113 | 21.3 | 0.3 |
| Syl2012_9_0_10 | 6271 | 121 | 8 | 65 | 778.5 | 1.9 |
| Population 2 |  |  |  |  |  |  |
| Syl2012_24_0_1 | 878 | 138 | 33 | 418 | 26.3 | 0.3 |
| Syl2012_24_0_2 | 654 | 38 | 6 | 33 | 106.6 | 1.1 |
| Syl2012_24_0_4 | 804 | 82 | 8 | 34 | 96.2 | 2.4 |
| Syl2012_24_0_7 | 2219 | 90 | 15 | 147 | 151.3 | 0.6 |
| Syl2012_24_0_10 | 2451 | 116 | 15 | 149 | 162.7 | 0.8 |
| **Mean (SD)** | **1628 (1775)** | **85 (37)** | **13 (8)** | **121 (114)** | **163 (222)** | **1 (1)** |
