# Supplementary material for: Effects of polyploidy on the coordination of gene expression between organellar and nuclear genomes in Leucanthemum Mill. (Compositae, Anthemideae)
Source: Ecol Evol. 2019 Jul 17;9(16):9100–10. doi: 10.1002/ece3.5455 (PMC6706232; doi:10.1002/ece3.5455)
Supplement: Supplementary file 5 [file ECE3-9-9100-s005.doc]

**Table S5.** Results of statistical test carried out in the present study on ploidy-dependent gene-copy and transcript numbers in three *Leucanthemum* species with diploid, tetraploid, and hexaploid chromosome numbers. Statistical tests performed are indicated as ^1)^ANOVA (assumptions: normal distribution + variance homogeneity), ^2)^non-parametric Kruskal-Wallis and Bonferroni-corrected Mann-Whitney-U tests; additional indices indicate whether ^3)^natural log transformation of data or ^4)^square-root transformation of data has been carried out in order to attain fulfilment of assumptions for the parametric ANOVA and post-hoc test.

|  |  | **Hypothesis accepted** | **Signif. differences between groups** |
| --- | --- | --- | --- |
| **Gene copy counts** | psbA/actin | ^1,3)^ H_A_(F = 10.910, df = 2, P = 0.00) | 2*x* > 6*x,* 4*x* > 6*x* |
|  | rbcL/actin | ^1,3)^ H_A_(F = 6.38, df = 2, P = 0.006) | 2*x* > 6*x* |
|  | psbO/actin | ^2)^ H_0_ (h = 0.567, df = 2, P = 0.753) | - |
|  | rbcS/actin | ^1,3)^ H_0_ (F = 0.144, df = 2, P = 0.867) | - |
|  | psbA/psbO | ^1,3)^ H_A_ (F = 9.49, df = 2, P = 0.001) | 2*x* > 6*x*, 4*x* > 6*x* |
|  | rbcL/rbcS | ^1,4)^ H_A_ (F = 3.681, df = 2, P = 0.039) | 2*x* > 6*x* |
| **Transcript copy counts** | psbA/actin | ^2)^ H_0_ (h = 1.295, df = 2, P = 0.523) | - |
|  | rbcL/actin | ^2)^ H_0_ (h = 3.625, df = 2, P = 0.163) | - |
|  | psbO/actin | ^2)^ H_0_ (h = 5.348, df = 2, P = 0.069) | - |
|  | rbcS/actin | ^1,3)^ H_A_ (F = 11.983, df = 2, P = 0.00) | 2*x* < 4*x*, 4*x >* 6*x* |
|  | psbA/psbO | ^2)^ H_0_ (h = 2.299, df = 2, P = 0.317) | - |
|  | rbcL/rbcS | ^1)^ H_A_ (F = 8.722, df = 2, P = 0.001) | 2*x* > 4*x*, 2*x* > 6*x* |
| **Transcript / gene copy number** | (cDNA psbA/actin)/(gDNA psbA/actin) | ^2)^ H_0_ (h = 1.887, df = 2, P = 0.389) | - |
|  | (cDNA rbcL/actin)/(gDNA rbcL/actin) | ^1,3)^ H_A_ (F = 4.127, df = 2, P = 0.028) | 2*x* < 4*x* |
|  | (cDNA psbO/actin)/(gDNA psbO/actin) | ^2)^ H_0_ (h = 5.363, df = 2, P = 0.068) | - |
|  | (cDNA rbcS/actin)/(gDNA rbcS/actin) | ^1,3)^ H_A_ (F = 13.017, df = 2, P = 0.000) | 2*x* < 4*x*, 4*x* > 6*x* |
|  | I (cDNA psbA/psbO)/(gDNA psbA/psbO) | ^2)^ H_0_ (h = 0.056, df = 2, P = 0.972) | - |
|  | I (cDNA rbcL/rbcS)/(gDNA rbcL/rbcS) | ^2)^ H_A_ (h = 6.296, df = 2, P = 0.043) | 2*x* > 4*x* |
| **Chloroplast number per cell** | guard cells | ^2)^ H_A_ (h = 104.276, df = 2, P = 0.000) | 2*x* < 6*x*, 4*x* < 6*x* |
|  | palisade parenchyma cells | ^2)^ H_A_ (h = 70.985, df = 2, P = 0.000) | 2*x* <4*x*, 2*x* < 6*x*,  4*x* > 6*x* |
|  | spongy parenchyma cells | ^1,4)^ H_0_ (F = 1.822, df = 2, P = 0.163) | - |
